# Ecological gradients driving the distribution of four Ericaceae in boreal Quebec, Canada

**DOI:** 10.1002/ece3.1476

**Published:** 2015-04-09

**Authors:** Nelson Thiffault, Pierre Grondin, Jean Noël, Véronique Poirier

**Affiliations:** 1 Direction de la recherche forestière, Ministère des Forêts, de la Faune et des Parcs du Québec2700 rue Einstein, Québec, G1P 3W8, Quebec, Canada; 2 Centre d’étude de la forêt, Faculté de foresterie, de géographie et de géomatique, Université Laval2405 rue de la Terrasse, Québec, G1V 0A6, Quebec, Canada

**Keywords:** Boreal forest, *Chamaedaphne calyculata*, ecological drivers, *Kalmia angustifolia*, landscape ecology, redundancy analysis, *Rhododendron groenlandicum*, *Vaccinium angustifolium*, *Vaccinium myrtilloides*

## Abstract

Understory species play a significant role in forest ecosystem dynamics. As such, species of the Ericaceae family have a major effect on the regeneration of tree species in boreal ecosystems. It is thus imperative to understand the ecological gradients controlling their distribution and abundance, so that their impacts can be taken into account in sustainable forest management. Using innovative analytical techniques from landscape ecology, we aimed to position, along ecological gradients, four Ericaceae found in the boreal forest of Quebec (Canada) (*Rhododendron groenlandicum, Kalmia angustifolia, Chamaedaphne calyculata*, and *Vaccinium* spp), to regionalize these species into landscape units relevant to forest management, and to estimate the relative importance of several ecological drivers (climate, disturbances, stand attributes, and physical environment) that control the species distribution and abundance. We conducted our study in boreal Quebec, over a study area covering 535,355 km^2^. We used data from 15,339 ecological survey plots and forest maps to characterize 1422 ecological districts covering the study region. We evaluated the relative proportion of each ericaceous species and explanatory variables at the district level. Vegetation and explanatory variables matrices were used to conduct redundancy, cluster, and variation partitioning analyses. We observed that ericaceous species are mainly distributed in the western part of the study area and each species has a distinct latitudinal and longitudinal gradient distribution. On the basis of these gradients, we delimited 10 homogeneous landscape units distinct in terms of ericaceous species abundance and environmental drivers. The distribution of the ericaceous species along ecological gradients is closely related to the overlaps between the four sets of explanatory variables considered. We conclude that the studied Ericaceae occupy specific positions along ecological gradients and possess a specific abundance and distribution controlled by the integration of multiple explanatory variables.

## Introduction

Understory vegetation plays a significant role in forest ecosystem dynamics (Gilliam 2007). Studies in many parts of the world have illustrated their impacts on soil properties (Heneghan et al. 2006) and nutrient cycling (Sharma and Raghubanshi 2009). Some understory herb and shrub species (e.g., *Pteridium aquilinum* [L.] Kuhn) can also act as a filter on tree species succession both prior and after major stand disturbances (Den Ouden 2000; Royo and Carson 2006), thus affecting tree recruitment and stand structure (Finegan 1984; Lorimer et al. 1994). Because of their multiple ecological roles, understory species are frequently used as ecological indicators and for forest classification purposes (Cajander 1926).

**Figure 1:**
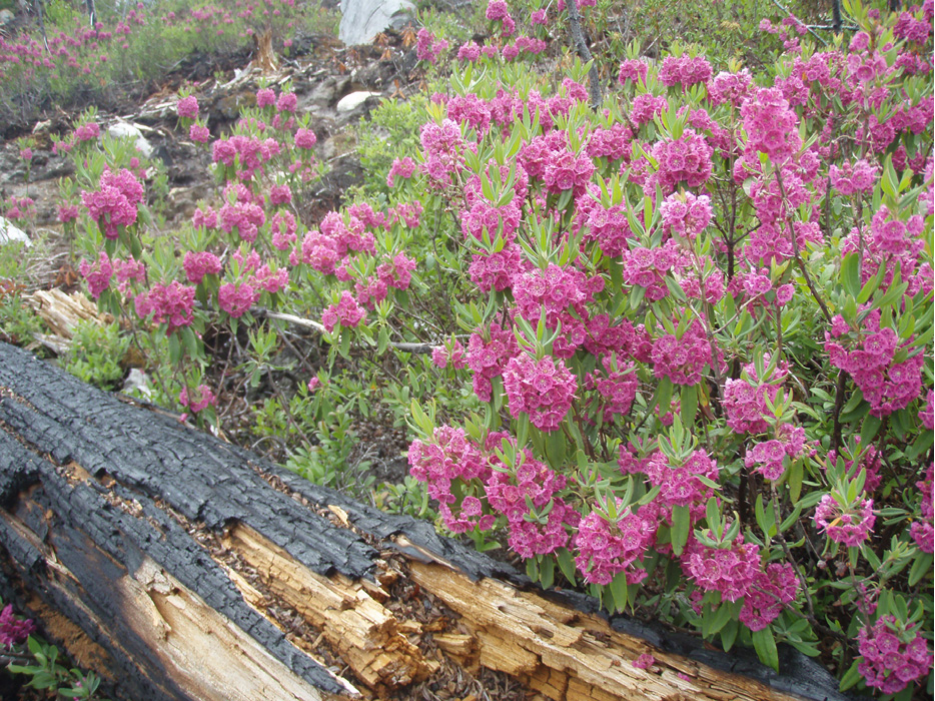
*Kalmia angustifolia* L., one of the four ericaceous species studied, regenerating on a recently burned site of northern Quebec, Canada.

Species of the Ericaceae family have major effects on the regeneration of tree species (Eşen et al. 2004; Nilsson and Wardle 2005). In northeastern America, the cover of ericaceous species such as *Kalmia angustifolia* L. (Fig.1), *Rhododendron groenlandicum* (Oeder) Kron & Judd, and *Vaccinium* sp. can rapidly increase following harvesting or wildfire events (Mallik 2003). They can induce ecosystem retrogression and create shrublands that persist for several decades (Damman 1971). The mechanisms responsible for the interference of Ericaceae on conifer establishment are numerous. For example, *Kalmia* and *Rhododendron* affect regenerating conifers by altering humus quality, by direct competition for nutrients, and through potential allelopathic effects (Inderjit and Mallik 1996, 2002; Thiffault et al. 2004; Joanisse et al. 2009). The “growth check” induced on regenerating conifers by ericaceous shrubs significantly reduces long-term forest productivity (de Montigny and Weetman 1990), and hence affects strategic forest planning, including calculation of annual allowable cuts (Thiffault et al. 2013).

The main factors driving the reproduction, growth, and morphology of east Canadian ericaceous species have been documented and reviewed (Hall et al. 1973; Hébert and Thiffault 2011). These species are relatively ubiquitous. Jobidon (1995) reports that *Kalmia* and *Rhododendron* are, respectively, found in 79% and 83% of the ecological sampling plots established in boreal Quebec (Canada). In comparison, *Rubus idaeus* L. and *Pteridium aquilinum* are found in 19% and 6% of the plots, respectively. Nonetheless, these ericaceous species respond locally to ecological drivers. For example, an assessment of three common ericaceous species conducted in a limited number of *Picea mariana* (Mill.) BSP stands of eastern Quebec have shown that locally, their presence is associated with canopy openness, tree species dominance, soil characteristics, and stand age (Laberge Pelletier 2007). In western Europe, ericaceous species distribution is also linked to site fertility (Økland 1996). A similar phenomenon is observed in eastern Europe (Shorohova et al. 2009).

In this study, we aimed to further investigate understory ericaceous species distribution at a large scale (>500,000 km^2^) and establish links between ecological gradients, ecosystem regionalization, and variation partitioning of landscape heterogeneity for this functional group (Turner 1989; Borcard et al. 1992; Økland 1996; Qian et al. 2003; McKenzie et al. 2011; Halvorsen 2012; Grondin et al. 2014). Specifically, our objectives were to use numerical ecology to (1) identify the ecological gradients along which four seemingly ubiquitous ericaceous species of northern Quebec (*Kalmia angustifolia*, *Rhododendron groenlandicum*, *Chamaedaphne calyculata* (L.) Moench., and *Vaccinium* sp.) are distributed, (2) regionalize the species distribution and four sets of explanatory variables (climate, disturbances, stand attributes, and physical environment) into homogeneous landscape units, and (3) estimate the relative importance of these four sets of explanatory variables in explaining landscape heterogeneity at the provincial and regional scales. The hypotheses that understory species can be regionalized at such a scale and that they respond to coherent combinations of drivers across scales remain to be tested. Our study follows those that have explained understory vegetation distribution using the integration of several sets of explanatory variables, at a scale compatible with landscape ecological classification and strategic forest planning (Qian et al. 2003). We directly address the need to conduct multiscale distribution modeling so that ecological processes operating at these scales can be better understood (Halvorsen 2012).

## Materials and Methods

### Study region

**Figure 2:**
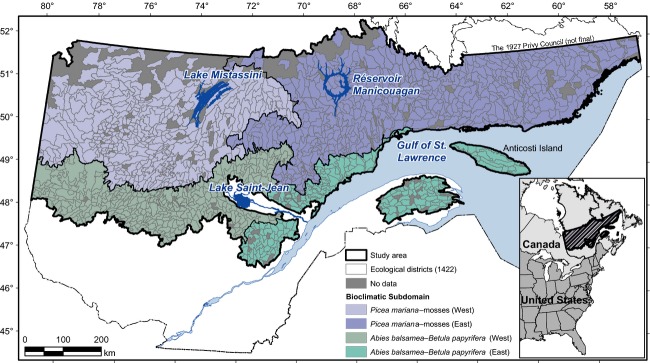
Location of the study region divided into ecological districts and bioclimatic subdomains (Saucier et al. 2009).

This study concerns a 535,355 km^2^ region of the circumboreal zone located in northern Quebec, covering two bioclimatic domains characterized by a continuous boreal forest zone (Fig.2; adapted from Saucier et al. 2009). Forest stands in the study region are mainly composed of softwood species (*Abies balsamea* (L.) Mill., *Picea mariana*, and *Pinus banksiana* Lamb.) and shade-intolerant hardwoods (mainly *Betula papyrifera* Marsh. and *Populus tremuloides* Michx.). Mean annual temperature varies from 0.8°C in the southern part of the study area to −1.0°C in the northern part. Mean annual precipitation varies according to a west–east gradient (1010–1127 mm). Geology consists of a core of old, massive Precambrian crystalline rocks (Laurentian and James Shield Regions; Bostock 1967). Surficial deposits are dominated by glacial, fluvial–glacial, and marine materials (Robitaille and Saucier 1998).

### Data sources

#### Ecological survey dataset

Data on the abundance of the four ericaceous species were extracted from a governmental ecological survey dataset. The survey was conducted between 1986 and 2000; 15,339 ecological sampling plots were established in our study area (Fig.2). Saucier et al. (1994) provide a detailed description of the sampling procedure. In summary, sampling plots consisted of circular 400 m^2^ plots distributed along 1.5–2 km sampling transects of 5–7 plots each. Each plot was described in terms of its geographical location (latitude, longitude, altitude) and physical features, including surficial deposit and drainage. Within the plots, percent cover of each plant species was visually estimated using the following classes: 6–25%, 26–40%, 41–60%, 61–80%, and >80%. Species with percent cover <6% were classified as 0%, <1%, or 1–5%. We converted these classes to the following values: 0%, 1%, 3%, 15%, 33%, 50%, 70%, and 90%. *Vaccinium angustifolium* Ait. and *V. myrtilloides* Michx. were grouped and treated at the genus level. Although these species are known to slightly differ in terms of site preference at the local scale, they share a similar transcontinental distribution, many morphological attributes and reproduction mechanisms (Bell et al. 2011). Moreover, species from the *Vaccinium* genus are known to naturally hybridize (Vander Kloet 1988), which complicates field identification. Hence, they are treated concomitantly in some North American silvicultural guides (Ontario Ministry of Natural Resources 1997; Beaudet et al. 2013).

The last major perturbation for each plot was determined by visual search of burned (fire) and logged stumps. The origin period was estimated based on the dominant tree age class; in case of doubt, two dominant trees were cored at the base to determine their age. From the dataset, we extracted data regarding tree species cover to determine the forest type based on the two most abundant species.

#### Forest survey dataset

We used geospatial databases developed from forest maps produced at the scale of 1:20,000 from aerial photographs. This forest information system is known as the *Spatial Information on Forest composition based on Tesserae (SIFORT)*. The maps produced between 1970 and 1980 represent the first decennial program, and those produced between 1980 and 1990, the second decennial program. A tessera corresponds to a segment of 15” of latitude and 15” of longitude (geographic coordinates) and covers 14 ha. Each tessera is characterized in terms of forest composition by superimposing its center on a forest map derived from the corresponding decennial forest inventory program (Pelletier et al. 2007). We extracted data regarding natural disturbances relative to the contemporary history (the last 150 years) of fires and insect outbreaks. The SIFORT geospatial database provided data for areas affected by spruce budworm (*Choristoneura fumiferana* [Clemens]), hemlock looper (*Lambdina fiscellaria* [Guen.]), windthrow, and natural fires. The number of years of infestation by spruce budworm and the frequency of fires 100 km^−2^ between 1938 and 1998 were determined from government archives. SIFORT also provided data on the proportion of land committed to agriculture and logging (human disturbances).

#### Ecological district dataset

Data from the ecological survey dataset and SIFORT were synthesized for each ecological district, a level of the hierarchical classification system in Quebec (Saucier et al. 2009). Ecological districts are delimited based on homogeneous physical environment, climate, and vegetation communities (Jurdant et al. 1977; Robitaille 1994). There are 1422 districts in the study region, and their mean area is 335 km^2^. Each district is described in a dataset, from which we extracted information about mean altitude and relative proportion of surficial deposits. The climate of each ecological district was characterized using the BioSIM simulator (Régnière et al. 2014). Climatic variables were estimated for the center of each district from a total of 51 meteorological stations located in the study region. Data from weather stations closest to each sampling location were used, after compensation for differences in latitude, longitude, and altitude using a combination of multiple linear regression and distance-weighting (Nalder and Wein 1998). Régnière and Bolstad (1994) and Régnière (1996) provide further descriptions of the approach used by the BioSIM tool. Other examples of its use in plant ecology include Andalo et al. (2005) and Ung et al. (2001). Normals were calculated for the 1961–1990 period (Régnière and St-Amant 2007) from 30 years of daily weather data (Environment Canada 2010).

### Data analysis

#### Autecology of the ericaceous species at the plot level

We used the data from the ecological survey plots to characterize the ericaceous species distribution along geographical gradients (latitude, longitude, altitude) and environmental categories (forest type, age class, origin, surficial deposit, and drainage). For each species, we restricted these descriptive analyses to plots with >25% cover, a subjective threshold we considered as minimal to represent a favorable growth environment and that would allow illustrating meaningful relations between the species and their environment. As a result, 1681 plots were used for *Kalmia*, 2828 for *Rhododendron*, 224 for *Chamaedaphne*, and 1112 for *Vaccinium*.

#### Species abundance and distribution at the district level

Using the same subset of plots, percent cover values were averaged by ecological district. The resulting means were mapped for the entire study zone and were used for all further analyses.

#### Ecological gradients and regionalization

Our analyses were based on two matrices describing the ecological districts using the variables combined from the various data sources. A first matrix (Y) contained the dependent variables, that is, the mean percent cover of the four ericaceous species at the district level. A second matrix (X) contained the explanatory variables at the district level, classified into four sets: forest types (15 variables), climate (eight variables), natural (nine variables) and human disturbances (two variables), and the physical environment (13 variables), for a total of 47 independent variables (Table1). Values for the forest types, physical environment, and disturbance variables were synthesized at the district level using their relative abundance within each district.

**Table 1:** Codes and description of the variables used in the study

Theme^1^	Code	Description
	CAL	*Cassandra calyculata*
	KAA	*Kalmia angustifolia*
	RHG	*Rhododendron groenlandicum*
	VAAM	*Vaccinium angustifolia* and *Vaccinium myrtilloides*
Forest types	Ab	Relative area for *Abies balsamea* forest type
	Ba	Relative area for *Betula alleghaniensis* forest type
	Bp	Relative area for *Betula papyrifera* forest type
	Bp-Ab	Relative area for *Betula papyrifera*–*Abies balsamea* forest type
	Bp-Pm	Relative area for *Betula papyrifera*–*Picea mariana* forest type
	H	Relative area for heathland
	Pb	Relative area for *Pinus banksiana* forest type
	Pm	Relative area for *Picea mariana* forest type
	Pm-Ab	Relative area for *Picea mariana* and *Abies balsamea* forest type
	Pt	Relative area for *Populus tremuloides* forest type
	Pt-Ab	Relative area for *Populus tremuloides*–*Abies balsamea* forest type
	Pt-Pm	Relative area for *Populus tremuloides*–*Picea mariana* forest type
	To	Relative area for *Thuja occidentalis* forest type
	Bog	Relative area for nonforested wetland
Physical environment	Alti	Mean altitude (m)
	D_1A	Relative area covered by thick till (more than 1 m)
	D_1AR	Relative area covered by thin till (less than 1 m)
	D_R	Relative area covered by rock
	D_2	Relative area covered by juxtaglacial deposits
	D_4GA	Relative area covered by glacio-lacustrine fine-textured (clay) surficial deposit
	D_7	Relative area covered by organic deposits
	D_8	Relative area covered by thick alterites (more than 1 m)
	D_8AR	Relative area covered by thin alterites (less than 1 m)
	D_wa	Relative proportion of area covered by water
	Ele	Average elevation in meters over a distance of 1 km
	P_def	Relative area covered by slopes over 15%
Climate	Ari	Aridity index (cm)
	Dwfc	Number of consecutive days without freezing
	Eva	Evapotranspiration (cm)
	Gdd	Annual number of growing degree-days
	Mat	Mean annual temperature (°C)
	Prect	Total precipitation (rain and snow) during the year (mm)
	Precu	Useful precipitation (rain) (mm)
	Vpd	Vapor pressure deficit (total daily deficit (mbar) from months of June to August)
Disturbances	Ag	Relative area covered by agriculture
	Br	Relative area burned by recent fires (natural and human)
	Ft	Relative area covered by forest tent (*Malacosoma disstria*) outbreak
	Hf	Relative area covered by human fires
	Hl	Relative area covered by hemlock looper (*Lambdina fiscellaria*)
	Log	Relative area covered by recent logging
	Sbom	Relative area covered by mild spruce budworm (*Choristoneura fumiferana*) outbreak
	Sbos	Relative area covered by severe spruce budworm (*Choristoneura fumiferana*) outbreak
	Sbon	Number of years of infestation by spruce budworm during the period 1938–1998
	O1700	Plots originating from fires before 1870
	O1880	Plots originating from fires between 1870 and 1900
	O1900	Plots originating from fires between 1900 and 1920
	O1920	Plots originating from fires after 1920

1 See methods for data sources.

**Figure 3:**
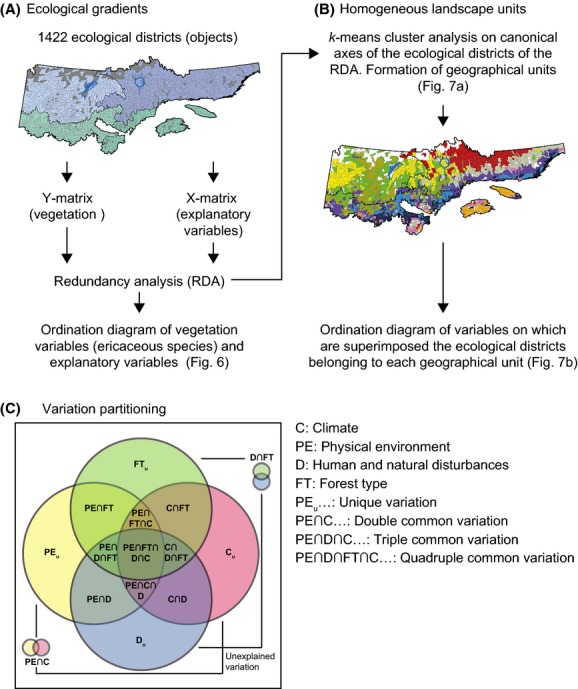
Methodology flowchart to (A) identify the ecological gradients controlling the distribution of four ericaceous species in Quebec, Canada; (B) define homogeneous landscape units on the basis of ericaceous species distribution and their ecological drivers; and (C) partition the relative importance of the climate, disturbance, forest type, and physical environment variables controlling species distribution. The Venn diagram illustrates the variation partitioning among the four sets of explanatory variables. The rectangle represents the total variation of species distribution, with 15 fractions of explained variation.

We submitted the matrices to a redundancy analysis (RDA) (Fig.3A) (Borcard et al. 2011; Legendre and Legendre 2012). The objective of a RDA is to extract and summarize the variation in a set of response variables (identified as the Y-matrix) that can be explained by a set of explanatory variables (identified as the X-matrix). In a RDA, we thus regress multiple response variables on multiple explanatory variables and then subject the matrix of the fitted values of all response variables to a principal components analysis (Borcard et al. 2011). The RDA results in an ordination diagram that summarizes the patterns of variation in the Y-matrix of dependent variables that can be explained by the X-matrix of explanatory variables. Prior to the RDA, the variables of the Y-matrix were subjected to a Hellinger transformation to give less weight to abundant ericaceous species and to preserve an ecologically meaningful distance among sites in the ordination (Legendre and Gallagher 2001). We conducted the RDA with the *Vegan* package of R (R Development Core Team 2010). The RDA results (canonical axes) were used to produce an ordination diagram on which we located the ericaceous species and the explanatory variables selected using a forward selection approach.

To define homogeneous geographical units based on ericaceous species composition and explanatory variables, separate *k-means* cluster analyses were carried out on four canonical axes of the ecological districts (Fig.3B). The final number of clusters was based on the Calinski–Harabasz criterion (Borcard et al. 2011) and on empirical knowledge of the descriptive and explanatory variables for the study region (Robitaille and Saucier 1998; Grondin et al. 2007).

Variation partitioning was used to quantify the relative importance of the sets of explanatory variables on ericaceous cover variability (Borcard et al. 1992; Dray et al. 2012; Legendre et al. 2012). Variation partitioning consisted of a series of partial RDAs between the Y- and X-matrices. Fifteen partial RDAs were required to complete a Venn diagram for the four sets of explanatory variables (Fig.3C). Variation partitioning was conducted using the *Varpart* package of R, following the steps proposed by Borcard et al. (2011), as adapted to our study: (1) Hellinger transformation for the Y-matrix; (2) creation of a separate X-matrix for each set of explanatory variables (X-Climate, X-Disturbance, X-Physical environment, X-Forest types); (3) creation of parsimonious X-matrices by running four separate partial RDA-based forward selections, using an adjusted R^2^; (4) variation partitioning of Y-matrix using the four parsimonious X-matrices; and (5) tests of significance (by permutations) on the 16 testable fractions of variation obtained from the analyses (15 of these defined the explained variation, among which 11 were common to two, three, or four sets of explanatory variables; high values of common variation indicate a strong overlap between vegetation and explanatory variables). All testable fractions were significant. We carried out five partitioning analyses. Whereas the first analysis encompassed the entire study region (provincial scale), the others (regional scale) separately considered the western, eastern, southern, and northern parts of the study region, divided based on the bioclimatic domains (north–south division) or subdomains (east–west division) (Fig.2).

## Results

### Species autecology and distribution

**Figure 4:**
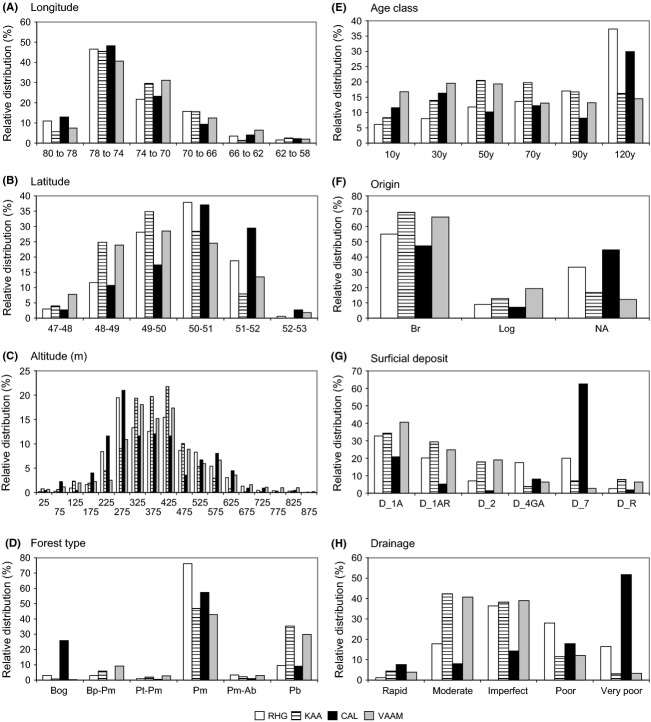
Relative distribution (%) of the sampling plots with >25% cover of each species, for a selection of explanatory variables. (A), (B) and (C) relate to the geographical location; (D), (E) and (F) relate to the forest stand; (G) and (H) relate to the soil environment. For (E), age class values are as follows: 10 (0–20 years); 30 (21–40 years); 50 (41–60 years); 70 (61–80 years); 90 (81–100 years); and 120 (>100 years). For (F), NA stands for “Natural”, which means that a specific origin was not available. Refer to Table1 for other variable description and codes.

**Figure 5:**
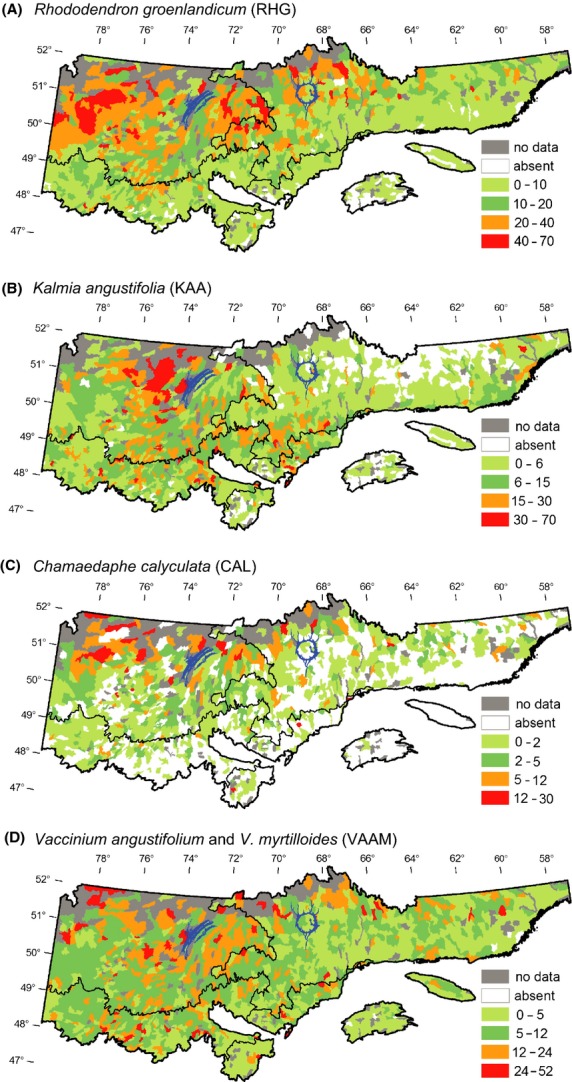
Distribution and relative abundance (%) by ecological district (*n* = 1422) of four ericaceous species in the study region, based on data from the ecological survey plots (*n* = 15,339). Refer to Figure2 for the general location of the study region, geographic landmarks, and scale. (A) *K. angustifolia*; (B) *R. groenlandicum*; (C) *C. calyculata;* (D) *V. angustifolium* and *V. myrtilloides*.

The four ericaceous species are mainly distributed in the western subdomains of the closed boreal forest (Fig.4). They are scarce between longitudes 58°–66° W, occasional between 66° and 70°W, and abundant west of 70°W. *Rhododendron* is the most abundant species among the four studied (Fig.5). Its mean percent cover is usually >10%. *Kalmia* presents a mean percent cover similar to that of *Rhododendron* (>6%). However, districts with a mean percent cover of *Kalmia >*15% are scarcer than for *Rhododendron*. They are mainly located west of Lake Mistassini (Fig.5). *Chamaedaphne* presents a lower mean percent cover than the other studied species (always <30%; Fig.5). As observed for *Rhododendron* and *Kalmia*, *Chamaedaphne* is most abundant in the *Picea mariana*–mosses bioclimatic domain. *Vaccinium* species (*augusifolium* and *myrtilloides*) present moderate mean percent cover (5–24%) in a large proportion of the ecological districts west and east of *Réservoir Manicouagan* (Fig.5). Contrary to *Kalmia* though, *Vaccinium* is relatively abundant in the eastern part of the study region, notably between longitudes 62° and 66°W (Gaspé Peninsula and northern shore of the St. Lawrence River) (Fig.4).

### Ecological gradients

**Figure 6:**
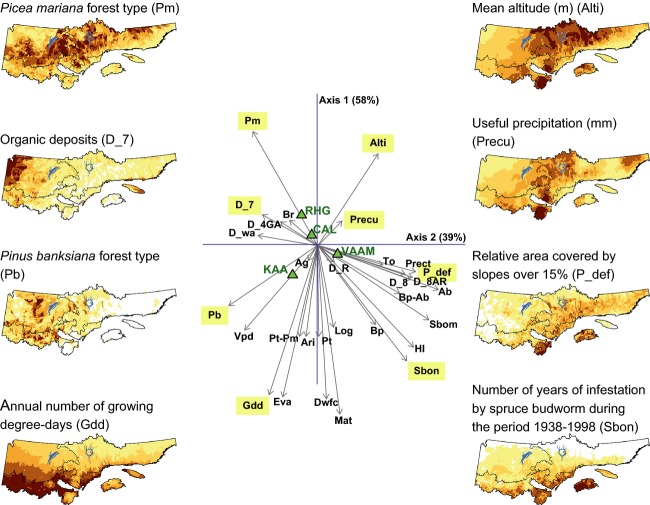
Ordination diagram resulting from a redundancy analysis (RDA) performed on the dependent (Matrix Y, describing the mean percent cover of the four ericaceous species at the district level) and explanatory variables (Matrix X, describing four sets of explanatory variables: forest types, climate, disturbances, physical environment) of the study area. Maps of selected variables (forward selection) are included for interpretation purposes. Axis 1 expresses the latitudinal gradient and axis 2, the longitudinal gradient. Refer to Figure2 for the general location of the study region, geographic landmarks, and scale and to Table1 for variable description and codes.

Ecological gradients representative of the study region were estimated based on the ordination presented in Figure6. Axis 1 is positioned vertically as it describes a latitude gradient, whereas axis 2 is horizontal and expresses a longitudinal gradient. The latitude gradient is a stronger driver of ericaceous species distribution than the longitude gradient. On the one hand, the latitude gradient is driven by variables representative of the southern part of the study region, such as growing degree-days and the number of years of spruce budworm infestations. On the other hand, this gradient is influenced by conditions representative of the northern part of the study zone, such as the abundance of *Picea mariana* stands and high altitudes. The longitudinal gradient extends from the western part of the study region, well stocked in *Pinus banksiana* stands (southwest) and organic deposits (northwest), to its southeastern part, dominated by steep slopes and many years of spruce budworm outbreaks.

Among the studied species, *Kalmia* and *Vaccinium* have the strongest association with the southern part of the study region (the *Abies balsamea–Betula papyrifera* bioclimatic domain). In addition to the gradients described above, *Kalmia* is closely associated with regions characterized by a high vapor pressure deficit. Environmental variables associated with *Vaccinium* are characteristic of steep slope terrains, namely thick or thin surficial deposits, and stands dominated by *Abies balsamea* or *Thuya occidentalis* L.

*Rhododendron* and *Chamaedaphne* are mostly associated with the northern regions of the gradients (the *Picea mariana*–mosses bioclimatic domain). Burned areas are found in the same area of the ordination diagram, being closely associated with *Picea mariana* and *Pinus banksiana* stands. High altitude districts are located in the upper right quadrant of the ordination, being associated with the *Rhododendron*/*Picea mariana* stands of the central boreal zone and the *Vaccinium*/*Abies balsamea* stands of southeastern boreal Quebec.

### Regionalization

**Figure 7:**
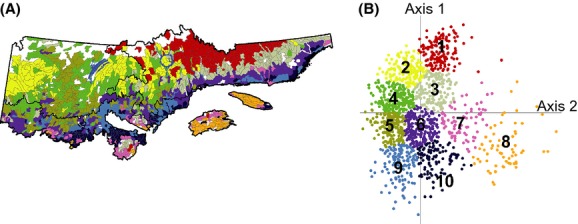
(A) Regionalization of the study region into 10 homogeneous landscape units (HLU). Colors are used to differentiate the units and do not reflect species abundance. Black lines separate the bioclimatic subdomains. Refer to supporting informations for a detailed description of the HLU and to Figure2 for the general location of the study region, geographic landmarks, and scale. (B) Ordination diagram from a redundancy analysis (RDA) showing the position of ecological districts (*n* = 1422) along the ecological gradients characterizing the study region. Colors are used to differentiate the 10 HLU and correspond to those used in panel (A). Ordination is the same as in Figure6.

Cluster analyses based on the canonical axes of the ecological districts led to the definition of 10 homogeneous landscape units (Fig.7) that can be described using the dominant ericaceous species.

#### Units dominated by Rhododendron

Units 1, 2, and 3 share dominance by *Rhododendron*. In these units, *Vaccinium* is subdominant, whereas *Chamaedaphne* and *Kalmia* are not abundant. These units are located in the northern part of the study area (Fig.7A). *Rhododendron* abundance diminishes from units 2 to 1, and 3. The soil is dominated by till, with the exception of the western part of units 2, where organic and clay deposits are abundant. Rock is frequent in the eastern part of the study area (northern shore of the St. Lawrence River), in units 1 and 3. Overall, *Picea mariana* and old forests are abundant, whereas *Pinus banksiana* is scarcely found. *Picea mariana*–*Abies balsamea* stands are well represented in units 1, 2, and 3, mainly in the center and eastern parts of the *Picea mariana*–mosses bioclimatic domain. Altitude is variable, but unit 1 (central part of the territory) presents high altitudes (631 m). Unit 3, the most maritime of all, has a low cover of ericaceous species and high occurrence of *Picea mariana*–*Abies balsamea*-dominated stands, as well as *Abies balsamea*-dominated stands. Unit 3 is characteristic of the northern shore of the St. Lawrence River and of the >800 m altitude of the Laurentian Mountains and *Monts-Valin* (c. 48° 38′N; 70° 52′W).

#### Units dominated by Kalmia and Rhododendron

Units 4, 5, and 6 share dominance by *Kalmia* and *Rhododendron*. *Vaccinium* species are subdominant, and *Chamaedaphne* is not abundant. Units 4 and 5 are located in the western part of the study area (Fig.7A), west of *Réservoir Manicouagan*. Ericaceous species are relatively abundant. *Picea mariana* is dominant, whereas *Pinus banksiana* is subdominant. Wildfires from the 1920 period are abundant. *Pinus banksiana* is well represented in unit 5, where *Kalmia* reaches its maximum abundance. Unit 6 mainly comprises ecological districts in which nonforested areas are well represented. This unit mainly characterizes the maritime border of the northern shore of the St. Lawrence River, although it is also present in the western *Abies balsamea–Betula papyrifera* bioclimatic subdomain. Ericaceous species are less abundant in this unit than in the previous ones, with the three species having comparable low abundance. On the northern shore of the St. Lawrence River, the unit is mainly composed of open woodlands with abundant rock outcrops. Within the western part of the domain, this unit mainly characterizes ecological districts affected by harvesting activities.

#### Units dominated by Vaccinium

These units (7, 8) are characterized by a low abundance of ericaceous species. Their total cover is generally ≤5%. The understory is mainly dominated by *Latifoliaea*. These units are found in maritime regions of the *Abies balsamea–Betula papyrifera* bioclimatic domain (Fig.7A). *Abies balsamea*-dominated stands and *Betula papyrifera*-dominated stands are well represented. *Betula alleghaniensis* Britt. and *Thuya occidentalis* are also present. The Gaspé Peninsula is distinct with high altitudes and a rough topography (abundant slope >15%). The Anticosti Island is a vast, low-altitude calcareous plateau.

#### Units dominated by Kalmia and Vaccinium

Units 9 and 10 share dominance by *Kalmia* and *Vaccinium*. *Rhododendron* is subdominant. These units are almost exclusively restricted to the *Abies balsamea–Betula papyrifera* bioclimatic domain (Fig.7A). The ericaceous species are less abundant than in the units located within the *Picea mariana*–mosses bioclimatic domain. *Kalmia* and *Vaccinium* are found in diverse forest types, like *Picea mariana* stands, *Pinus banksiana* stands, *Betula papyrifera* stands, and *Populus tremuloides* stands. Wildfires of the 1920 period are abundant. Unit 10 is of higher altitude and has a rougher topography than unit 9. These territories are characterized by harvesting activities and spruce budworm breakouts.

### Relative importance of the explanatory variables

Analyses of variation partitioning were used to estimate the contributions of each set of explanatory variables alone (e.g., C_u_, unique variation due to climate) and in combination with others (e.g., C_c_, common variation involving climate) to the vegetation heterogeneity (Fig.3C). Total explained variation of the vegetation by the explanatory variables was relatively low (37.9%; Table2, upper part). However, the total relative proportion of the explained variation associated with each set of explanatory variables was relatively high (56.7–66.4%). The variation associated with unique fractions (e.g., C_u_) was low and always <10%. Rather, most of the variation was explained by common fractions (e.g., C_c_), mainly related to triple and quadruple combinations of explanatory variables (Table2). The quadruple interaction explained the highest proportion of the variation (21%).

**Table 2:** Relative proportion of variation (%) in the abundance of the ericaceous species (combined) explained by four sets of explanatory variables (climate [C], disturbances (natural and human) [D], physical environment [PE], and forest types [FT]). Partial redundancy analysis using Y- (ericaceous species cover) and X-(explanatory variables) matrices were used to estimate the total explained and unexplained variations. The explained variation is decomposed into 15 fractions as illustrated in Figure3C. Subscript “u” refers to unique variation fractions; subscript “c” refers to common variation fractions

Total variation	(%)
Explained	37.9
Unexplained	62.1
Relative proportion of explained variation (15 fractions)	
Unique variation	
C_u_	4.2
D_u_	2.1
PE_u_	5.5
FT_u_	6.8
Common variation	
PE∩D	3.4
PE∩C	12.1
C∩D	3.7
C∩FT	2.6
FT∩D	6.0
PE∩FT	5.0
PE∩C∩D	2.6
FT∩D∩C	10.8
PE∩FT∩C	7.1
PE∩FT∩D	7.1
PE∩FT∩D∩C	21.0
Explained variation	100.0
Sums of relative proportion of explained variation	
Total (t) unique relative variation	18.6
Total (t) common relative variation	81.4
Total relative variation by set	
C	64.0
D	56.7
PE	63.8
FT	66.4
Common (c) relative variation by set	
C_c_	59.8
D_c_	54.6
PE_c_	58.3
FT_c_	59.6

**Figure 8:**
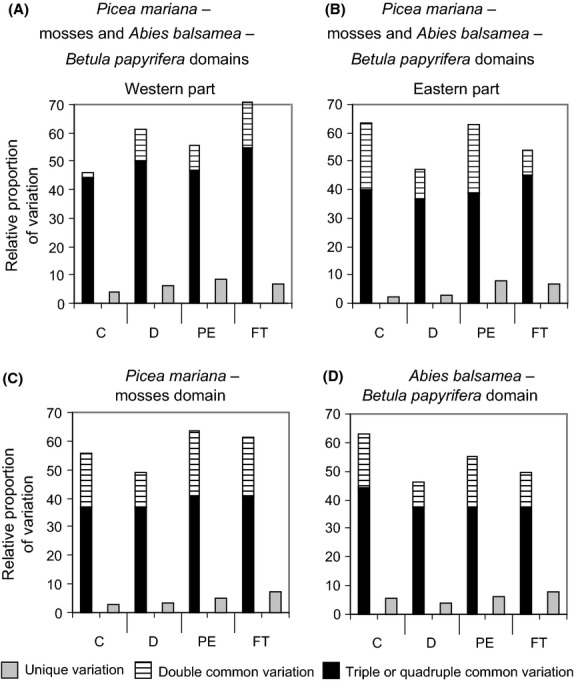
Synthetic view of the relative proportion of variation (%) in the abundance of the ericaceous species (combined) explained by four sets of explanatory variables (climate [C], disturbances (natural and human) [D], physical environment [PE], and forest types [FT]), separately considering the (A) western part of the study region, which includes the western parts of the *Picea mariana*–mosses and *Abies balsamea–Betula papyrifera* bioclimatic domains, (B) eastern part of the study region, which includes the eastern parts of the *Picea mariana*–mosses and *Abies balsamea–Betula papyrifera* bioclimatic domains, (C) northern part of the study region, corresponding to the entire *Picea mariana*–mosses bioclimatic domain, and (D) southern parts of the study region, corresponding to the entire *Abies balsamea–Betula papyrifera* bioclimatic domain (refer to Fig.2 for bioclimatic domain localization). The variation is defined by the common and unique fractions as illustrated in Figure3C.

Variation partitioning was also conducted separately on four sections of the study area (west, east, north, and south, based on the limits of the bioclimatic domains and subdomains; Fig.2) with the objective of characterizing the variations in the overlapping of sets at a lower scale than the whole territory (Fig.8). The analyses for the western part of the study region considered the components on the left-hand side of the ordination (Figs.7). These analyses revealed that the changes along units 2, 4, and 5 (the *Picea mariana*–mosses bioclimatic domain) toward units 9 and 10 (the *Abies balsamea–Betula papyrifera* bioclimatic domain) are mainly associated with changes in forest types as well as with natural and anthropogenic disturbances (Fig.8A). The northern part is characterized by infrequent fires (abundance of stands originating from the 1880 period) and abundance of *Picea mariana* stands. The southern part is defined by more frequent fires (1920 period) and numerous stands dominated by broadleaf and *Pinus banksiana* forest types. *Kalmia* and *Vaccinium* are the most abundant ericaceous species.

Analyses for the eastern part of the study region were based on elements from the right-hand side of the ordination (Figs.7). They informed on the changes from units 1, 3, and 6 (the *Picea mariana*–mosses bioclimatic domain) to units 7 and 8 (the *Abies balsamea–Betula papyrifera* bioclimatic domain). Results showed that old-growth forests are relatively abundant across this region, so changes in ericaceous species dominance are mainly associated with changes in the physical environment and climate (Fig.8B). Changes in the physical environment were expressed by many variables, for example, the abundance of alterites and slopes >15% in the *Abies balsamea* domain. The same was observed for climate, notably for the growing degree-days.

Analyses for the northern part of the study region were conducted to understand the changes from units 2, 4, and 5 (western *Picea mariana*–mosses bioclimatic subdomain) to units 1, 3, and 6 (eastern *Picea mariana*–mosses bioclimatic subdomain; Figs.7). We observed that changes are mainly related to the physical environment and forest types, with a certain effect of climate (Fig.8C). Altitude is among the physical environment variables that explain the changes. Forest types change from dominance by *Picea mariana*, *Pinus banksiana*, and a high percent cover of *Kalmia* to dominance by *Picea mariana, Abies balsamea*, and a high percent cover of *Rhododendron*.

Variation partitioning conducted for the southern part of the study region informed on the elements driving the changes from units 9 and 10 (western *Abies balsamea–Betula papyrifera* bioclimatic subdomain) to units 7 and 8 (eastern *Abies balsamea–Betula papyrifera* bioclimatic subdomain; Figs.7). Our results demonstrated that changes in ericaceous cover between these groups of units are mainly associated with changes in climatic variables; the other variable categories were subdominant (Fig.8D). Changes in climate were characteristic of the longitudinal gradient, such as the west*–*east reduction in the vapor pressure deficit.

## Discussion

Our study is in line with previous work grounded in numerical ecology that has defined ecological gradients for large territories, regionalizing vegetation along ecological gradients (e.g., ecological regions, homogeneous units), and quantified the overlap that exists between descriptive variables and sets of explanatory variables along ecological gradients (Ohmann and Spies 1998; Borcard et al. 2011; Legendre and Legendre 2012). Here, we applied numerical approaches to understand the distribution and abundance of four ericaceous understory species for which no clear relationship with large physical perturbation, forest type, and climatic gradients had previously been identified.

Ecological gradients describe the gradual changes in vegetation and explanatory variables through space. These changes define landscape heterogeneity (Milne 1991; Wagner and Fortin 2005). Many forest classification studies present local ecological gradients using toposequences illustrating forest types along altitude and drainage gradients (e.g., Gauvin and Bouchard 1983; Blouin and Berger 2005). Our study addresses landscape heterogeneity for a much larger territory (535,355 km^2^), which enables latitude and longitude gradients to be taken into account. The same gradients are used to elaborate ecological land classifications in many parts of the world, like southwestern USA (Ohmann and Spies 1998), Norway (Bakkestuen et al. 2008), and Quebec (Saucier et al. 2009). De Grandpré et al. (2014) have demonstrated how fire recurrence, cover types, moisture regime, and latitudinal gradients drive the large-scale distribution of understory species in boreal Quebec. Our results demonstrate that the same gradients drive the distribution of a more specific, seemingly ubiquitous group of understory species through their effects on environmental variables such as growing degree-days and the vapor pressure deficit. The large-scale impacts of these ecological drivers are consistent with the auto-ecological characteristics of ericaceous species documented at the local scale. For example, the association between *Rhododendron* cover and wild fire is coherent with its high morphological and physiological plasticity in response to changing light conditions, as demonstrated in controlled conditions (Hébert et al. 2011).

Ecosystem regionalization aims at segmenting ecological gradients and landscape heterogeneity into land portions of increasing homogeneity as scale became more and more detailed (e.g., from 1:500,000 to 1:250,000). The resulting cells can then be used to analyze ecological processes and forest stand dynamics at different levels of perception and integrate new knowledge into forest management tools. Ecological regionalization at provincial, national, or continental scales (performed at the biome level) are usually based on the links between vegetation and climate (Küchler 1964; Rowe 1972). Classification work conducted at smaller scales has shown that variables related to the physical environment, such as topography and surficial deposits, are important drivers of landscape structure (Hare 1950; Rowe 1962; Gerardin and Ducruc 1990). However, regionalization based on an a priori integration of vegetation data, climatic variables, the physical environment, and disturbances are scarce (Daubenmire 1936; Grimm 1984). Our ericaceous shrub regionalization is consistent with the classical forest ecological classification system (Saucier et al. 2009) as well as with the fire regime distribution (Ministère des Ressources naturelles du Québec 2013). It supports that these species alone constitute a faithful indicator of key ecosystems characteristics (Boulanger et al. 2013; Grondin et al. 2014), probably owing to their efficient regeneration strategy (Mallik 1993) coupled with their high trait plasticity (Hébert et al. 2011) to changing environments.

Variation partitioning provides the statistical means to quantify the relative importance of different sets of explanatory variables on vegetation variability (Borcard et al. 1992; Legendre et al. 2005; Dray et al. 2012; Halvorsen 2012). The proportion of explained variation was low in our study (37.9%), but consistent with results obtained in Norway (Økland and Eilertsen 1994) and the Russian Far East (Cushman and Wallin 2002). The relatively low *n* of our dataset (from 224 to 2828, depending on species) might explain this result. As observed in many studies that have used variation partitioning (Borcard et al. 1992 and following), our results nevertheless confirm the relative overlap and synchronism that exists between species abundance and explanatory variables; indeed, the variability in ericaceous cover was mainly explained by the combination of variables (double, triple, and quadruple), rather than by the sole effect of either set of variables (unique variation).

Our study is in line with those that have integrated numerous sets of variables describing both the habitat (climate, physical environment, disturbances) and forest vegetation (the forest type) (Qian et al. 2003). Here, we show that this last set of variables explains a similar proportion of the variability as that associated with the other sets. Dominant canopy trees, for example, *Populus tremuloides* vs. *Picea mariana*, directly affect the understory vegetation through the litter input and by modifying the microclimate, and indirectly via their effects on the humus-layer attributes (Qian et al. 2003; Arbour and Bergeron 2011). Our results support that there is a strong relation between forest types and understory species and that each association occupies a particular ecological niche (Hutchinson 1957; Légaré et al. 2001, 2004). They confirm that at the circumboreal scale, forests dominated by *Pinus*, *Picea,* or *Abies* are associated with specific understory species, including Ericaceae (Økland 1996; Saucier et al. 2009; Shorohova et al. 2009).

We have performed separate variation partitioning analyses for subregions within our study area (Ohmann and Spies 1998) and showed that within each subregion, the cover of seemingly ubiquitous ericaceous species is driven by specific combinations of variables (Fig.8). Our results inform that, for most parts of the study region, variation in ericaceous species cover is mainly driven by climate and the physical environment, in combination with other sets of variables (Fig.8B–D). This pattern can be exemplified by the eastern part of boreal Quebec (Fig.8B). The fire cycles are long in this region (Bouchard et al. 2008), and forest types are dominated by *Picea mariana–Abies balsamea*; these two sets of variables are thus relatively homogeneous at this scale. However, the zone is characterized by strong latitudinal climatic and physical environmental gradients that extends from the Gulf of St. Lawrence toward the interior of the province (Ministère des Ressources naturelles du Québec 2013). These two sets of variables are thus the main drivers of understory changes. Cushman and Wallin (2002) have also concluded that environmental conditions, particularly major elevation and physiographic gradients, are somewhat more important drivers than fire history in far East Russia.

In areas with short fire cycles and landscape characterized by a great diversity of forest types (e.g., *Pinus banksiana* vs. *Picea mariana* stands), such as the western part of boreal Quebec, our results rather indicate that variation in ericaceous species cover is mainly driven by natural disturbances and forest cover (Fig.8A). This important role of natural disturbances is in line with other studies that have investigated landscape heterogeneity (Heinselman 1973; Rowe and Scotter 1973). At the regional scale, Parisien and Sirois (2003) also identified wildfires as the main driver of vegetation changes in the James Bay region (northwestern Quebec, c. 50° 02′N; 80° 06′W).

## Conclusion

The distribution of seemingly ubiquitous species such as the ericaceous shrub *Rhododendron, Kalmia*, *Vaccinium,* and *Chamaedaphne* is heterogeneous and driven at the landscape level by interactions between stands (tree cover), physical environment, disturbances, and climatic variables. The patchy spatial pattern characterizing landscape heterogeneity is created by synchronous changes in vegetation and explanatory variables along environmental gradients. Our study illustrates the feedback that exists between landscapes patterns and ecological processes (McKenzie et al. 2011). The integrative approach used to evaluate how various drivers simultaneously control landscape heterogeneity was verified at both the canopy (Grondin et al. 2007, 2014) and understory levels (this study), at various spatial scales. Such studies could be extended to others groups of species (e.g., mosses) in the boreal forest, to evaluate how species functional traits influence these relations.

Our study is based on the use of large datasets accumulated over decades by governmental agencies for management and ecological classification purposes at the provincial scale. Our study is thus limited by many factors, such as the unequal (and sometime low) plot densities across ecological districts or the visual estimation of species cover performed by different survey teams over the years. These limits are, however, common to studies conducted at such a scale and do not prevent the identification of general gradients driving species distribution (e.g., Grondin et al. 2014). The maintenance of forest productivity and biodiversity, as influenced by ericaceous species, is a significant forestry issue in Quebec. The knowledge gained in this study now allows fine-tuning of the actual provincial ecological classification system. This information also provides the ecological basis for subdividing the study area into relatively homogeneous landscapes for finer-scale analyses, as well as for developing regional strategies regarding biological diversity, conservation, forest management, and the impact of global changes. This new ecological information will enable forest managers and silviculturists to assess the risk of ericaceous species dominance following harvesting or wild fires, depending on the region where they conduct forest activities (Thiffault et al. 2013). Our study is based on the use of powerful, yet descriptive statistical approaches. Further work should concentrate on the elaboration of predictive models of species distribution and abundance that could take into account future climate normals and management activities (Boulanger et al. 2013). Ultimately, this large-scale assessment of ericaceous species distribution will support the development of sustainable forest management strategies, which must imply maintaining the functions and structure of forest ecosystems (Burton et al. 2003).

## Supporting Information

**Table S1.** Description of the 10 homogeneous landscape units (HLU) in reference to vegetation variables. **Table S2.** Description of the 10 homogeneous landscape units (HLU) in reference to physical environment variables. **Table S3.** Description of the 10 homogeneous landscape units (HLU) in reference to climatic and disturbance variables.
