# Supplementary material for: Ecological gradients driving the distribution of four Ericaceae in boreal Quebec, Canada
Source: Ecol Evol. 2015 Apr 9;5(9):1837–53. doi: 10.1002/ece3.1476 (PMC4485965; doi:10.1002/ece3.1476)
Supplement: Supplementary file 1 [file ece30005-1837-sd1.docx]

Table S1. Description of the 10 homogeneous landscape units (HLU) in reference to vegetation variables. Ericaceous species values represent mean percent cover within the unit. Refer to Table 1 for other variable description and units.

|  |  | Ericaceous species | | | |  | Broadleaf dominated forest types | | | | | | |  | Conifer dominated forest types | | | | |  | Others | |
| --- | --- | --- | --- | --- | --- | --- | --- | --- | --- | --- | --- | --- | --- | --- | --- | --- | --- | --- | --- | --- | --- | --- |
| HLU | Nb^1^ | RHG | KAA | CAL | VAAM |  | Ba | Bp | Bp-Ab | Bp-Pm | Pt | Pt-Ab | Pt-Pm |  | Ab | Pb | Pm | Pm-Ab | To |  | Bog | H |
| 1 | 145 | 13 | 0 | 1 | 8 |  | 0 | 1 | 0 | 1 | 0 | 0 | 0 |  | 1 | 0 | 39 | 16 | 0 |  | 3 | 22 |
| 2 | 146 | 25 | 5 | 2 | 8 |  | 0 | 0 | 1 | 2 | 0 | 0 | 1 |  | 1 | 3 | 43 | 11 |  |  | 17 | 5 |
| 3 | 149 | 8 | 3 | 1 | 6 |  | 0 | 2 | 1 | 3 | 1 | 0 | 1 |  | 5 | 1 | 27 | 24 | 0 |  | 3 | 12 |
| 4 | 205 | 22 | 13 | 3 | 11 |  | 0 | 1 | 1 | 2 | 1 | 0 | 2 |  | 1 | 9 | 38 | 6 | 0 |  | 12 | 5 |
| 5 | 190 | 13 | 15 | 1 | 10 |  | 0 | 1 | 1 | 3 | 1 | 0 | 2 |  | 2 | 16 | 28 | 5 | 0 |  | 10 | 3 |
| 6 | 193 | 6 | 8 | 0 | 6 |  | 0 | 4 | 2 | 4 | 3 | 0 | 2 |  | 5 | 5 | 22 | 16 | 0 |  | 5 | 8 |
| 7 | 80 | 2 | 1 | 0 | 4 |  | 2 | 8 | 10 | 4 | 2 | 1 | 2 |  | 18 | 0 | 11 | 12 | 1 |  | 4 | 4 |
| 8 | 65 | 0 | 1 | 0 | 2 |  | 2 | 8 | 8 | 2 | 2 | 2 | 2 |  | 30 | 0 | 8 | 13 | 1 |  | 2 | 2 |
| 9 | 136 | 6 | 12 | 1 | 9 |  | 0 | 4 | 2 | 3 | 6 | 1 | 5 |  | 4 | 15 | 15 | 7 | 0 |  | 5 | 7 |
| 10 | 113 | 2 | 6 | 0 | 5 |  | 1 | 13 | 4 | 4 | 6 | 1 | 5 |  | 6 | 6 | 15 | 7 | 0 |  | 3 | 7 |

^1^ Number of ecological districts.

Table S2. Description of the 10 homogeneous landscape units (HLU) in reference to physical environment variables. Refer to Table 1 for variable description and units and to Table 2 for the number of ecological districts in each HLU.

| HLU | Alti | D_1A | D_1AR | D_R | D_2 | D_4GA | D_7 | D_8 | D_8AR | D_wa | Ele | P_def |
| --- | --- | --- | --- | --- | --- | --- | --- | --- | --- | --- | --- | --- |
| 1 | 632 | 50 | 16 | 17 | 5 | 0 | 2 | 0 | 0 | 8 | 78 | 21 |
| 2 | 432 | 35 | 10 | 7 | 4 | 12 | 18 | 0 | 0 | 8 | 48 | 11 |
| 3 | 484 | 35 | 23 | 21 | 6 | 2 | 3 | 0 | 0 | 8 | 80 | 26 |
| 4 | 383 | 34 | 12 | 8 | 6 | 12 | 13 | 0 | 0 | 11 | 43 | 9 |
| 5 | 384 | 36 | 14 | 8 | 9 | 5 | 10 | 0 | 0 | 13 | 46 | 10 |
| 6 | 355 | 23 | 22 | 21 | 6 | 7 | 5 | 0 | 0 | 9 | 68 | 20 |
| 7 | 489 | 37 | 29 | 11 | 5 | 1 | 4 | 2 | 1 | 5 | 101 | 36 |
| 8 | 419 | 21 | 15 | 7 | 1 | 0 | 3 | 31 | 18 | 1 | 111 | 39 |
| 9 | 319 | 26 | 21 | 20 | 12 | 0 | 4 | 0 | 0 | 8 | 67 | 19 |
| 10 | 383 | 30 | 29 | 16 | 9 | 0 | 3 | 1 | 1 | 6 | 85 | 26 |

Table S3. Description of the 10 homogeneous landscape units (HLU) in reference to climatic and disturbance variables. Refer to Table 1 for variable description and units and to Table 2 for the number of ecological districts in each HLU.

|  | Climate | | | | | | | |  | Disturbances | | | | | | | | | | | | |
| --- | --- | --- | --- | --- | --- | --- | --- | --- | --- | --- | --- | --- | --- | --- | --- | --- | --- | --- | --- | --- | --- | --- |
| HLU | Ari | Dwfc | Eva | Gdd | Mat | Prect | Precu | Vpd |  | Ag | Br | Ft | Hf | Hl | Log | Sbom | Sbos | Sbon | O1700 | O1880 | O1900 | O1920 |
| 1 | 1.0 | 146 | 33 | 774 | -2.7 | 1015 | 325 | 924 |  | 0.0 | 7 | 0 | 0 | 0 | 1 | 0 | 1 | 0 | 61 | 15 | 14 | 6 |
| 2 | 1.1 | 157 | 36 | 1009 | -1.6 | 983 | 329 | 1119 |  | 0.0 | 6 | 0 | 1 | 0 | 3 | 0 | 2 | 1 | 56 | 16 | 11 | 13 |
| 3 | 1.0 | 160 | 35 | 924 | -1.1 | 1165 | 341 | 974 |  | 0.0 | 6 | 1 | 1 | 1 | 7 | 3 | 0 | 2 | 50 | 14 | 14 | 12 |
| 4 | 1.3 | 162 | 37 | 1097 | -0.9 | 969 | 321 | 1179 |  | 0.1 | 8 | 1 | 3 | 0 | 7 | 1 | 0 | 2 | 36 | 16 | 16 | 22 |
| 5 | 1.5 | 168 | 38 | 1188 | -0.2 | 982 | 322 | 1233 |  | 0.4 | 6 | 1 | 13 | 1 | 12 | 2 | 4 | 2 | 19 | 13 | 16 | 30 |
| 6 | 1.6 | 172 | 38 | 1156 | 0.3 | 1070 | 319 | 1140 |  | 0.5 | 2 | 2 | 8 | 1 | 17 | 6 | 0 | 4 | 29 | 14 | 15 | 20 |
| 7 | 1.3 | 171 | 38 | 1143 | 0.5 | 1224 | 358 | 1108 |  | 0.0 | 1 | 2 | 2 | 2 | 22 | 21 | 0 | 8 | 16 | 11 | 16 | 23 |
| 8 | 1.7 | 177 | 39 | 1120 | 1.1 | 1165 | 314 | 1092 |  | 0.0 | 2 | 1 | 4 | 3 | 19 | 25 | 1 | 11 | 10 | 10 | 16 | 29 |
| 9 | 1.8 | 177 | 40 | 1263 | 0.8 | 1027 | 311 | 1213 |  | 0.4 | 5 | 2 | 20 | 1 | 23 | 4 | 5 | 5 | 13 | 10 | 14 | 24 |
| 10 | 1.7 | 177 | 39 | 1261 | 1.0 | 1093 | 326 | 1202 |  | 0.1 | 2 | 3 | 10 | 2 | 22 | 14 | 5 | 8 | 12 | 7 | 13 | 27 |
